# Technological potential of *Bacillus megaterium* in Cacao bean fermentation

**DOI:** 10.1007/s10123-025-00775-z

**Published:** 2026-03-13

**Authors:** Laura Sabrina Ortiz Galeano, Andrés Sandoval Lozano, Angélica Sandoval - Aldana, María Denis Lozano - Tovar

**Affiliations:** 1 Faculty of Agronomical Engineering, Universidad del Tolima, Barrio Santa Helena Parte Alta, Ibagué, Tolima Colombia; 2 Department of Agronomy, María de Maeztu Excellence Unit DAUCO.ETSIAM, Córdoba, España; 3 Corporación Colombiana de Investigación Agropecuaria (AGROSAVIA), Centro de Investigación Nataima, Km9, Espinal-Ibagué, Tolima Colombia

**Keywords:** Cacao fermentation, CCN51 cacao, Degree of fermentation, Microbiological starter, Microbiota

## Abstract

During the fermentation of cacao beans, a succession of microorganisms, including yeasts, lactic acid bacteria, acetic acid bacteria, and Bacillus, are involved in the generation of aroma and flavor precursors. These microbial consortia have been extensively studied, except for the genus *Bacillus*. Accordingly, this work aimed to isolate and select *Bacillus* isolates and evaluate their effect on the fermentation process of CCN51 genotype cacao beans. The isolates were obtained from a fermentation process using native cacao material. The isolates were selected based on growth performance and biological activity assays, including the production of pectinases, proteases, citrate lyases, and antifungal activity. Subsequently, their effect as a starter culture on the CCN51 genotype beans was evaluated. The isolates were individually evaluated at a concentration of 7 × 10^6^ CFU/mL in a proportion of 1% starter culture (v/w) relative to the cacao mass. Temperature, pH, acidity, fermentation index, and cut test were monitored, showing higher fermentation index values (up to 1.5) and a reduction of insufficiently fermented beans (from 36% in the control to 13–20%) in inoculated treatments. The evaluation of the effect of *Bacillus* isolate on cacao fermentation indicated that the *B. megaterium* isolate improved the sensorial quality of cacao beans, decreased their bitterness and astringency, and highlighted flavors such as floral, fruity, nutty, and cane sugar-malt. It also registered antifungal activity against mycotoxin-producing fungi. It can be concluded that *Bacillus* has biotechnological potential as a producer of enzymes of interest for cacao processing and the food industry.

**Supplementary Information:**

The online version contains supplementary material available at 10.1007/s10123-025-00775-z.

## Highlights

- Beans fermented with *Bacillus megaterium* exhibited a more favorable sensory profile.
- *B. megaterium* increased nutty, floral, and fruity flavors in cacao beans.
- Cacao beans inoculated with *B. megaterium* showed a better fermentation index.
- *Bacillus megaterium* showed antifungal activity against *Aspergillus* spp.

**Supplementary Information:**

The online version contains supplementary material available at 10.1007/s10123-025-00775-z.

## Introduction

The *Theobroma cacao* CCN51 genotype stands out due to its productivity, disease resilience, and auto-compatibility. However, CCN51 is not well accepted in the market because of its high astringency and bitterness (Navia and Pazmiño 2012). Another challenge in cacao bean quality is the presence of mycotoxins, mainly aflatoxins (Afs) and ochratoxin A (OTA), which represent a global concern (Delgado et al. 2021). When fermentation conditions are inadequate, fungi from the genera *Aspergillus* and *Penicillium* can colonize cacao beans and produce secondary metabolites that pose serious health risks (Veras et al. 2023). Therefore, identifying strategies to optimize cacao bean fermentation conditions is important.

Fermentation is an essential process for developing the organoleptic properties of cacao beans, including aroma and flavor (Porras et al. 2019). During cacao fermentation, multiple microorganisms and processing conditions interact to drive these changes (Schwan and Wheals 2004).

Several studies have identified microbial succession and its role in cacao fermentation, which typically lasts between 5 and 7 days. Yeasts dominate the initial stage (0–48 h), breaking down pectins, glucose, and sucrose while producing ethanol and CO₂ (Lima et al. 2011). The most common species include *Saccharomyces cerevisiae*, *Pichia kudriavzevii*, and *Hanseniaspora opuntiae* (Díaz et al. 2022). Subsequently, lactic acid bacteria (LAB) become predominant (24–72 h), contributing to the production of more than 85% of lactic acid, ethanol, acetic acid, and glycerol, with *Lactobacillus plantarum* and *Lactobacillus fermentum* being the most prevalent species (Schwan 1998; Lefeber et al. 2011; Papalexandratou et al. 2013; Vuyst and Weckx 2016). In the following phase (48–96 h), oxidation occurs as acetic acid bacteria metabolize ethanol into acetic acid. Finally, increased temperature and oxygen availability during the later stages of fermentation (> 48 h) stimulate the growth of *Bacillus* (Li et al. 2022).

Since the 20th century, starter cultures have been implemented in the cacao bean fermentation process to ensure that the final product, i.e., chocolate, has consistent characteristics and an enhanced sensory profile (Díaz and Vuyst 2022).

Studies on the inoculation of *B. amyloliquefaciens* under both aerobic and anaerobic conditions have demonstrated significant concentrations of ethylphenyl acetate (floral aroma) and ethanol (alcoholic note). Under anaerobic conditions, it also produces benzaldehyde, which imparts an almond-like aroma, and benzophenone, contributing to an herbal aroma (Tigrero et al. 2022). *Bacillus subtilis* and *Pichia kudriavzevii* were evaluated in co-culture and individually to determine what starting culture could be the most effective. While the two species were inhibited when worked in co-culture, as a single isolate, *B. subtilis* generated aromas that the yeast *Pichia kudriavzevii* was not able to produce, such as 1-butanol and 3-methyl, 1-butanol and 2-methyl, and 2,3-butanediol. These compounds express malty and chocolate, fruity and sweet aromas (Ouattara et al. 2020). At the same time, the presence of *Bacillus* in cacao beans has been associated with volatile compounds, such as acetoin, acetic acid, isovaleric acid, phenol, limonene, benzyl alcohol, and phenethyl alcohol (Mota-Gutiérrez et al. 2018).

Regarding the improvement of bean quality in terms of safety, certain *Bacillus* strains, such as *Bacillus thuringiensis* ATCC 10,792, exhibit fungicidal and fungistatic activity (Kadjo et al. 2023). Cedeño et al. (2023) reported that *B. subtilis* 31BMC and *B. licheniformis* E-44 strains significantly reduced the growth of *Aspergillus* spp. and *Penicillium* spp., inhibiting their development by 74.43% to 81.52% after three days. Therefore, this study aimed to isolate *Bacillus* from cacao fermentations and evaluate their effect on the sensory profile of cacao beans to develop a starter culture to enhance their quality and safety.

## Materials and methods

### Biologic material

The microorganisms were obtained from fermentation processes under common cacao producer conditions in the “Los Pasos” village in Guamo, Tolima, Colombia. They were isolated on a modified de Man, Rogosa and Sharpe (MRS) culture medium, consisting of 2% powdered milk without sugar, 0.4% yeast extract, 1.0% peptone, 1 mL/L Tween 80, 0.2% dipotassium phosphate, 0.5% sodium acetate, 0.3% ammonium sulfate, 0.02% magnesium sulfate, 0.005% manganese (II) sulfate, 2% agar, 0.2% trisodium citrate, 10% tomato juice, and 100 mg/L nystatin (Sánchez et al. 2011). The pH was adjusted to 5.6 before autoclaving. Plates were incubated at 30 °C for 8 days under aerobic conditions. Presumptive isolates were purified by successive streaking, and Gram staining was subsequently performed for preliminary identification.

### Selection of *Bacillus* isolates to improve the quality and safety of cacao beans

The *Bacillus* isolates were subjected to growing tests with 10% high glucose concentration, 5% ethanol, and a temperature of 50 °C. The control was cultured in an MRS medium and supplemented with each factor mentioned above for the test. The microorganism suspension was prepared in a 0.85% saline solution with 1 × 10^8^ CFU/mL concentration. Then, 10 µL of the suspension was placed on four points of the Petri dish with the respective culture medium and incubated at 30 ± 2 °C for 96 h (Lozano-Tovar et al. 2020). Afterward, the *Bacillus* growing area was registered with the Imagej Program. The data obtained was analyzed with an ANOVA, and the medium difference was calculated with Fisher’s LSD multiple range test at 0.5% using the Infostat and GraphPad Prism programs.

### Characterization of *Bacillus *isolates

#### Pectinase production

For pectinase production, 50% Cacao Pulp Medium Agar was used in 9 cm diameter Petri dishes. The medium was inoculated with 10 µL in four spots of the dish and incubated at 30 ± 2 °C for 48 h until the colonies grew to around 3 mm in radius. The dish that had the isolates was flooded with Lugol (1.0 g of iodine and 5.0 g of potassium iodide in 330 mL of H_2_O) to determine the halos (clarity zones) due to the degradation of the pectin of the medium by total pectinases produced by the organism (Ouattara et al. 2008).

#### Citrate consumption

Citrate consumption was evaluated in a Simmons Citrate solid Medium **(**trisodium citrate 0.2%, sodium chloride 0.5%, dipotassium phosphate 0.1%, ammonium dihydrogen phosphate 0.1%, magnesium sulfate 0.02%, bromothymol blue 0.08%, and agar 1.5%). The final medium pH was adjusted to 6.9 before autoclaving (Yao et al. 2017). A positive test is confirmed by growth with a color change from green to intense blue along the slant.

#### Protease production

For this test, the Milk Agar culture medium (meat peptone 0.5%, yeast extract 0.3%, skimmed milk 0.1%, and agar 1.2%) and the Casein Agar culture medium (meat peptone 0.5%, yeast extract 0.3%, skimmed milk 0.1%, and agar 1.2%) were used. The microorganism suspensions were prepared with saline solution at 0.85%, adding the microorganism until reaching a murkiness similar to MacFarland’s No. 2 tube. Then, 10 µL of microbial solution was inoculated in four points of the Petri dishes and incubated for 48 h at 30 ± 2 °C. Proteolytic activity was determined according to the change in color of the medium from white to transparent (Ferrero 1995).

#### Evaluation of the antifungal effect of *Bacillus *extracts on the mycelia growth of mycotoxin-producing fungi

Crude extracts were independently obtained from each *Bacillus* isolate. *Bacillus subtilis* GTBM2.19, *B. subtilis* GTBM4.67, *B. subtilis* GTBM7.101, *B. subtilis* GHALE2R2T2-2, and *Bacillus megaterium* GHAA2R3T1 were cultured for 72 h in HS liquid medium (2% glucose, 0.3% peptone, 0.5% yeast extract, 1.5% CaCO₃, and 1.2% agar) (Yao et al. 2017). Cultures were centrifuged at 10,000 rpm for 15 min, and the cell-free supernatants were collected. Supernatants were concentrated by air-drying at 28 ± 2 °C and resuspended in a volume ten times lower than the initial volume (Lozano-Tovar et al. 2023). The final concentration of the crude extracts was standardized by determining protein content using the Bradford method (Bradford 1976), with bovine serum albumin as standard at 595 nm. Extracts were sterilized by sequential filtration through 5.0, 0.45, and 0.20 μm filters.

For protein quantification, 40 µL of extract and 1.2 µL of Bradford reagent were mixed, incubated for 10 min, and measured at 595 nm using a Thermo Scientific™ G10S UV–Vis spectrophotometer. Non-fermented culture medium was used as control.

The antifungal activity was evaluated at a concentration of 25% (v/v) in Potato Dextrose Agar (PDA) medium. Fungal isolates of the genera *Aspergillus* and *Penicillium* were obtained from cacao fermentation under field conditions. The isolates were identified based on macroscopic and microscopic characteristics and maintained on PDA at 30 °C until analysis at the Microbiology Laboratory of the Nataima Research Center (Agrosavia). Conidia from two Aspergillus spp. isolates and one Penicillium spp. isolate were harvested using a 0.01% Tween 80 solution, and the suspensions were adjusted to 1 × 10⁶ conidia/mL. PDA plates were supplemented with the extracts, and a mycelial plug (5 mm diameter) was placed at the center of each plate. Plates without extract were used as controls. All assays were performed in triplicate. After incubation at 30 °C, radial mycelial growth was measured, and the percentage of mycelial growth inhibition was calculated relative to the control.

Mycelial growth inhibition (%) was calculated as: [(Dc − Dt)/Dc] × 100, where Dc is the colony diameter in the control and Dt is the diameter in the treatment.

#### Molecular identification of the isolates selected for the microbiological starter

##### DNA extraction

The DNA was extracted from pure cultures using the DNeasy UltraClean Microbial Kit (QIAGEN, Germany), following the manufacturer’s instructions. Then, it was quantified using a Nanodrop spectrophotometer (ND1000 Thermo Fisher Scientific, Milan, Italy).

##### Amplification

The bacterial DNA was amplified by PCR targeting the 16 S rRNA gene using primers 337 F (GACTCCTACGGGAGGCWGCAG), 518 F (CCAGCAGCCGCGGTAATACG), 800R (TACCAGGGTATCTAATCC), and 1100R (GGGTTGCGCTCGTTG) (Sánchez et al. 2022). The PCR products were purified and subsequently sequenced for taxonomic identification.

##### Identification

The analysis was performed on the Phylogeny.fr platform and comprised the following steps. Alignment of sequences with T-Coffee (v13.45.0.4846264) using the following pair-wise alignment methods: the 10 best local alignments (Lalign_pair) and an accurate global alignment (slow_pair). After alignment, ambiguous regions (i.e., containing gaps, poorly aligned, or both) were removed with Gblocks (v0.91b) using the following parameters: (i) minimum length of a block after gap cleaning: 10; (ii) no gap positions were allowed in the final alignment; (iii) all segments with contiguous non-conserved positions bigger than 4 were rejected; and (iv) minimum number of sequences for a flank position: 85%. The phylogenetic tree was reconstructed using the neighbor-joining method implemented in “Neighbor” included in the PHYLIP package (v3.696). Distances were calculated using FastDist. Graphical representation and edition of the phylogenetic tree were performed with TreeDyn (v198.3).

The PCR was carried out with 6.25 µL of Taq master mix solution, 0.25 µL of reverse primer, 0.25 µL of forward primer 20 µM, 50 ng of DNA template, and sterilized water free of nucleases, under the following conditions: an initial denaturalization phase at 94 °C for 3 min, followed by 30 cycles at 72 °C for 90 s, and finally, an extension at 72 °C for 10 min. The PCR products were visualized by electrophoresis using 2 µL of the reaction mixture on a 1% agarose gel stained with GelGreen^®^ (Biotium, USA) and documented using a UV transilluminator equipped with a UVITEC filter. Purified PCR products were sent to Macrogen Inc. (Seoul, Republic of Korea) for Sanger sequencing using the same primers employed for PCR amplification.

##### **Effects of the***Bacillus***starter culture on cacao quality**

The effect of the selected isolates was tested using two techniques: micro-fermentation and macro-fermentation. The isolates that performed best on the physical, chemical, and sensory profiles were assessed for this assay. Five *Bacillus* isolates, GTBM4.67, GTBM7.95, GTBM7.101, GHAA2R3T1, and GHALE2R2T2-2, were evaluated by micro-fermentation and two isolates, GHAA2R3T1 and GHALE2R2T2-2, by macro-fermentation.

##### Inoculum preparation

The selected isolates were cultured in PDA due to the rapid growth of *Bacillus* in this medium. After 48 h of incubation at 30 ± 2 °C, the *Bacillus* biomass was established per isolate, and suspensions with 0.85% saline solution were prepared. The concentrations of the treatments were adjusted to 7 × 10^6^ CFU/mL. The treatments were added to the cacao pulp in a 1% proportion (volume/weight).

##### Micro-fermentation

**Fig. 1:**
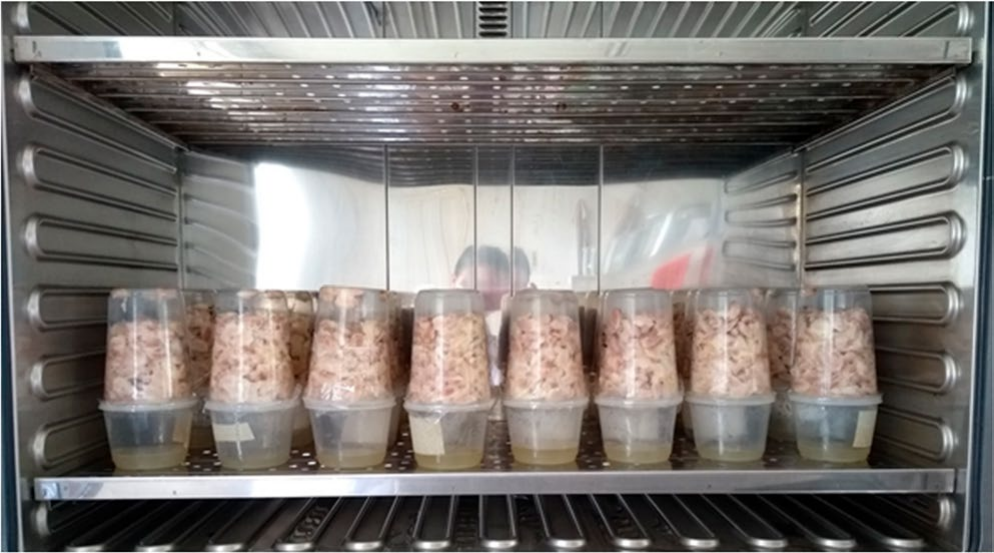
Cacao micro-fermentation process under controlled conditions

Only ripe cacao pods were harvested, washed with detergent, and sterilized with sodium hypochlorite at 1%. Then, they were rinsed and sprayed with 70% alcohol. The beans were removed from the pods in laminar flow cabinets, and the mass was homogenized, storing 900 g of the beans in plastic containers. These were adapted with holes in a closed system, allowing the drainage of exudates generated during the first 48 h of fermentation under anaerobic conditions. In this study, the micro-fermentation technique was defined as the controlled fermentation of small cacao masses under laboratory conditions, as described below. The containers were distributed inside an incubator using a completely randomized design. During the first day, the temperature was 35 °C (anaerobic phase), and after the third day, the microorganisms were added, and the mass was mixed. In this way, the system was no longer closed, and the temperature increased to 45 °C (aerobic phase) until the end of fermentation at day 7. For each experimental replicate, 900 g of cacao mass was used for a total of 2,700 g of cacao mass of the CCN51 genotype per treatment. The treatment not inoculated with microorganisms was used as a control (Fig. 1).

### Macro-fermentation

The sterilization and fermentation conditions were the same as those used for the micro-fermentation test. An amount of 10 kg of cacao beans was used in each fermenter with a leachate drainage system.

### Evaluation of the physical and chemical variables

#### pH and titratable acidity

A sample of 4 g of cacao beans was separated from the shell and ground with a mortar and pestle, adding 20 mL of distilled water. Then, the sample was filtered, and 10 mL of each solution was taken and labeled. NaOH at 0.01 N was added until reaching a final pH of 7.0 ± 0.02 (Wang 2022). The data was registered daily in triplicate during fermentation.

#### Cut test

Ten beans were randomly taken every 24 h and dried for five days. The beans were cut longitudinally with a scalpel to analyze them internally and determine the color changes and cracking; in this way, the beans were classified based on the NTC 1252 regulation as (i) insufficiently fermented, (ii) slightly fermented, or (iii) well fermented (Norma Técnica Colombiana ICONTEC, 2003). Hence, the percentage of beans in each category was registered.

#### Fermentation index

A sample of 0.2 g of cacao beans representative of the cut test was weighed and placed in dark Falcon tubes and mixed with 20 mL of a solution composed of 96% ethanol and 37% HCl (97:3, v/v), corresponding to a final HCl concentration of 1.11% (v/v). The mixture was homogenized for 20 min at 10,000 rpm and 4 °C. The supernatant was collected, and the absorbance was measured with a UV–VIS spectrophotometer at 460 and 530 nm (Eyamo et al. 2016).

#### Organoleptic quality of cacao beans

For the sensory profile, 250 g of cacao beans were collected in triplicate on the sixth and seventh day of fermentation from the micro- and macro-fermentation tests. The beans were dried for five days at 30 °C under shade. Once dried, they were toasted at 120 °C for 40 min and ground using a KitchenAid^®^ food processor. When the nibs took a liquid consistency (chocolate liquor), they were placed in a mold and stored at 4 °C until the sensory analysis was performed. A trained cacao sensory evaluation panel consisting of 4 panelists at the Nataima Research Center of Agrosavia carried out the sensory analysis of the cacao beans. Four specialized cacao tasters or evaluators integrated it. The panelists included in the study are part of the official cocoa sensory evaluation panel of the Nataima Research Center of Agrosavia, which was established based on training provided by the cocoa bean quality group of FEDECACAO (National Federation of Cacao Farmers), following the World Medical Association’s Code of Ethics (Declaration of Helsinki) for experiments involving humans (Criollo et al. 2023).

For this analysis, the samples were heated at 40 °C until their liquid state was recovered, and then they were distributed to the panel for evaluation. The panel used the following intensity scale: absent (0), low (1–2), medium (3–5), high (6–8), and very high (9–10) to assess nine attributes: acidity, bitterness, astringency, fruity, floral, raw, nutty, cane sugar-malt, and cacao (Quintana and Gomez 2011).

## Results

### Selection of *Bacillus* isolates to improve the quality and safety of cacao beans

**Fig. 2:**
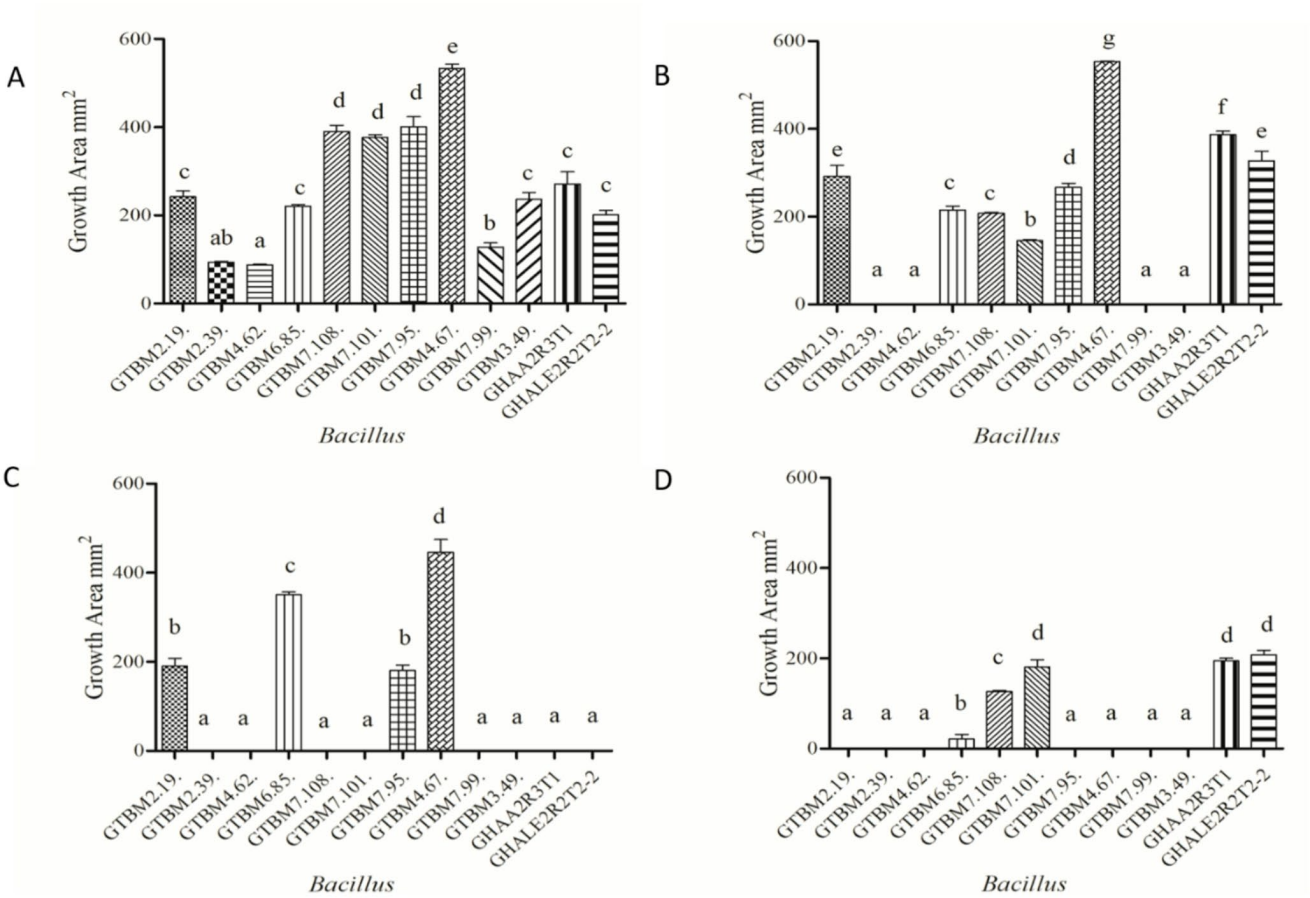
Growth tests for Bacillus isolates. Growth of isolates in the Man, Rogosa and Sharpe (MRS) medium (**A**), growth of isolates in 10% glucose-enriched medium (**B**), growth of isolates in 5% ethanol-enriched medium (**C**), and growth of isolates at 50 °C (**D**). Bars sharing the same letter are not significantly different, whereas bars with different letters indicate significant differences according to Fisher’s LSD test (*p* < 0.05)

Ten isolates of the *Bacillus* genus were obtained and codified as GTBM2.19, GTBM2.39, GTBM2.49, GTBM4.62, GTBM4.67, GTBM6.85, GTBM7.95, GTBM7.99, GTBM7.101, and GTBM7.108. Further, two other isolates, GHAA2R3T1 and GHALE2R2T2-2, were obtained from the working collection of the Nataima Research Center of Agrosavia (Table 1). These isolates were subjected to growth tests in MRS under different conditions. All isolates showed significant differences. However, five isolates exhibited the highest growth, with isolate GTBM4.67 showing the greatest colony surface area of 553 mm² on MRS medium (Fig. 2A), 553 mm^2^ in the medium enriched with 10% glucose (Fig. 2.B), and 446 mm^2^ in the medium enriched with 5% ethanol (Fig. 2.C).

**Table 1:** Isolates identified, obtained from Cacao fermentation processes in Tolima, Colombia

Accession	Isolate name		Species	
PX457896		GTBM2.19		*B. subtilis*
PX457916		GTBM4.67		*B. subtilis*
PX457915		GTBM7.95		*B. subtilis*
PX457914		GTBM7.101		*B. subtilis*
PX457910		GTBM7.108		*B. subtilis*
Not deposited (((Agrosavia collection)		GHAA2R3T1		*B. megaterium*
Not deposited (((Agrosavia collection)		GHALE2R2T2-2		*B. subtilis*

When the isolates were tested at high concentrations of glucose (10% glucose-enriched medium), the GTBM4.67 showed the highest growth (553 mm^2^), followed by GHAA2R3T1 (386 mm^2^). Four isolates (GTBM2.39, GTBM2.49, GTBM4.62, and GTBM7.99) were not able to grow on the enriched medium with 10% glucose (Fig. 2.B), so these were discarded as starter cultures. On the other hand, isolates GTBM2.19, GTBM4.67, GTBM6.85, GTBM7.95, GTBM7.101, GTBM7.108, GHAA2R3T1, and GHALE2R2T2-2 displayed a similar growth to the control (Fig. 2.A). The growth of isolates GTBM2.19, GTBM6.85, GHAA2R3T1, and GHALE2R2T2-2 was stimulated by the addition of glucose to the medium (Fig. 2B).

In the high ethanol (5%) concentration growth test, only four isolates GTBM2.19, GTBM4.67, GTBM6.85, and GTBM7.95) grew. However, isolate GTBM4.67 stands out with the highest growth (445 mm^2^) (Fig. 2.C).

In the growth tests at a temperature of 50 °C, only five isolates were able to grow. However, isolates GHAA2R3T1 and GHALE2R2T2-2 stood out as they showed the highest growth under this temperature condition and were statistically different from the other isolates (Fig. 2.D).

### Characterization of *Bacillus* isolates

**Fig. 3:**
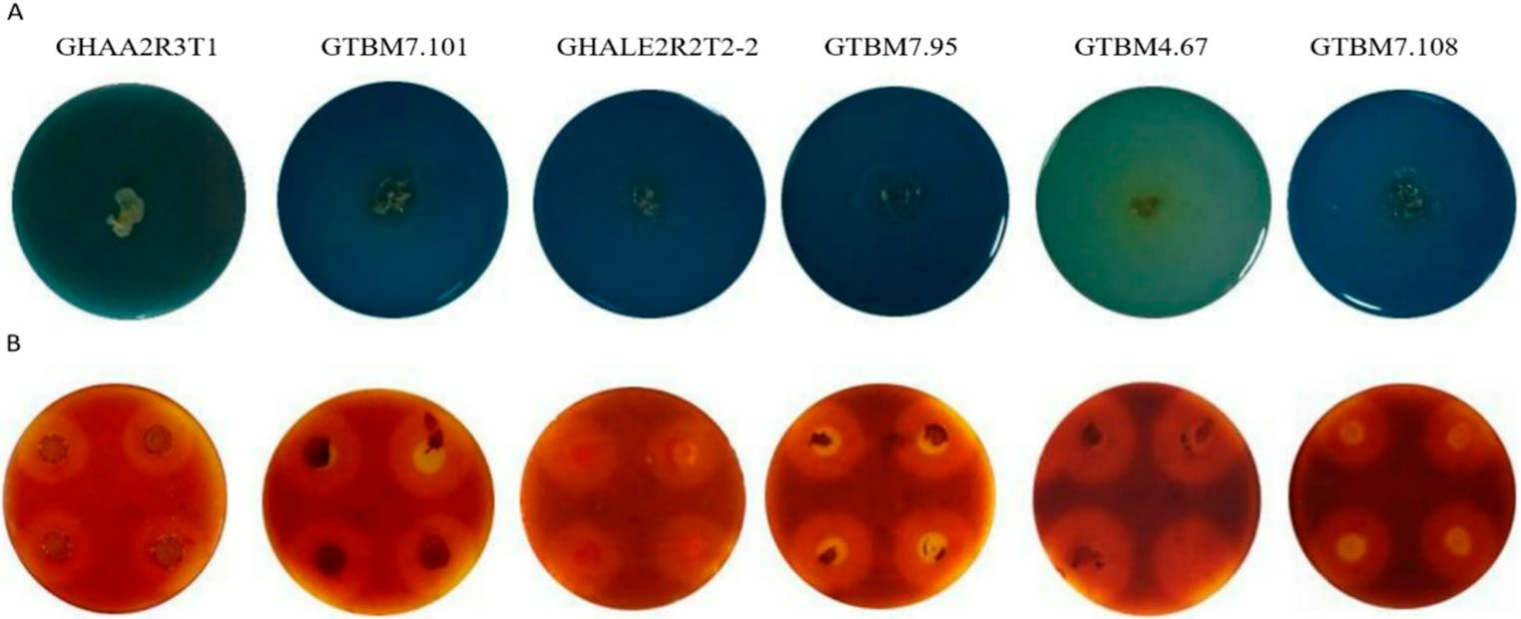
Characterization of *Bacillus* isolates. Simmons Citrate Solid Medium positive test is confirmed by growth with a color change from green to intense blue (**A**); Production of pectinases (pectinolytic activity) observed as halos (196.0 to 825.5 mm^2^) in 50% Cacao Pulp Medium Agar (**B**)

All the *Bacillus* isolates evaluated on Simmons Citrate Medium were positive for citrate lyase. Furthermore, all isolates exhibited pectinase production, evidenced by the formation of clear halos on 50% Cacao Pulp Medium Agar, with halo areas ranging from 196.0 to 825.5 mm² (Table 2; Fig. 3). Protease production was also confirmed, as the isolates formed clear halos on Casein Agar medium.

**Table 2:** Characterization of *Bacillus* isolates according to the growth and biological activity tests

Isolate	Pectinase halo (mm^2^)	Citrate lyase	Protease
GTBM2.19	636.3 ± 16.6 c	Positive	Positive
GTBM2.39	0 ± 0 f	Positive	Negative
GTBM3.49	676.3 ± 14.2 bc	Positive	Positive
GTBM4.62	701.5 ± 11.2 b	Positive	Positive
GTBM4.67	807.5 ± 24.3 a	Positive	Positive
GTBM6.85	0 ± 0 f	Negative	Negative
GTBM7.95	733.0 ± 37.6 b	Positive	Positive
GTBM7.99	0 ± 0 f	Negative	Negative
GTBM7.101	825.5 ± 51.3 a	Positive	Positive
GTBM7.108	694.3 ± 13.5 bc	Positive	Positive
GHAA2R3T1	508.0 ± 22.9 d	Positive	Positive
GHALE2R2T2-2	196.0 ± 15.1 e	Positive	Positive

Different letters indicate significant differences according to Fisher’s LSD test (*p* < 0.05). Values sharing the same letter are not significantly different

### Evaluation of the antifungal effect of *Bacillus* crude extracts on the mycelial growth of mycotoxin-producing fungi

**Fig. 4:**
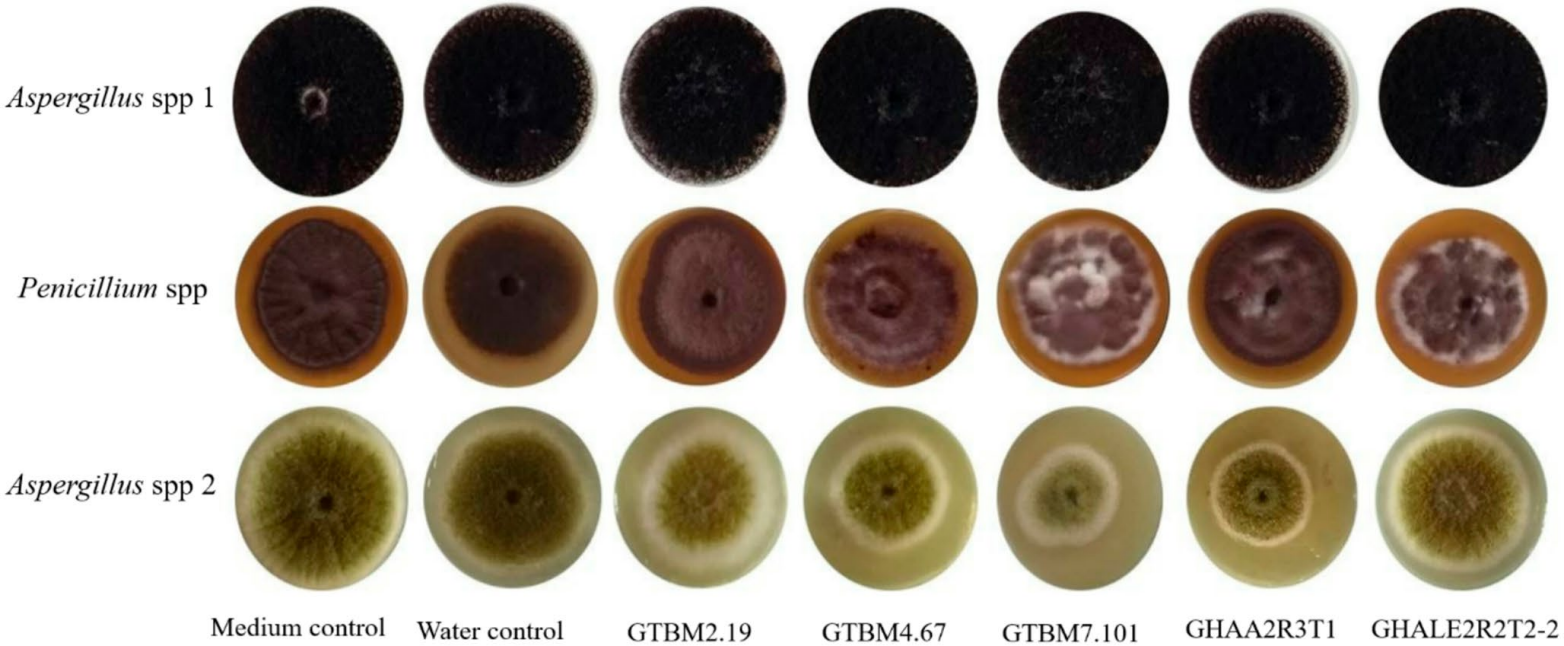
Evaluation of crude *Bacillus* extracts in inhibiting three isolates of filamentous fungi

When the *Bacillus* crude extracts of isolates GTBM2.19, GTBM4.67, GHAA2R3T1, GTBM7.101, and GHALE2R2T2-2 were added at a concentration of 25%, the growth of *Aspergillus* spp. 2 was inhibited (Fig. 4) from 32 to 48%. However, isolates GTBM7.101 and GHAA2R3T1 registered the highest inhibition, i.e., 44% and 48%, respectively (Table 3). The highest protein concentration was also recorded in these isolates.

**Table 3:** Percentage of Inhibition of crude *Bacillus* isolates extracts on *Aspergillus* spp 2 according to the percentage of protein in the extracts

Bacillus isolates	Inhibition (%)	Protein (µg/mL)*
GTBM2.19	32	0.20
GTBM4.67	37	0.09
GHAA2R3T1	48	0.21
GTBM7.101	44	0.58
GHALE2R2T2-2	0	0.14

* crude extracts

### Molecular identification of the isolates selected for the microbiological starter

**Fig. 5:**
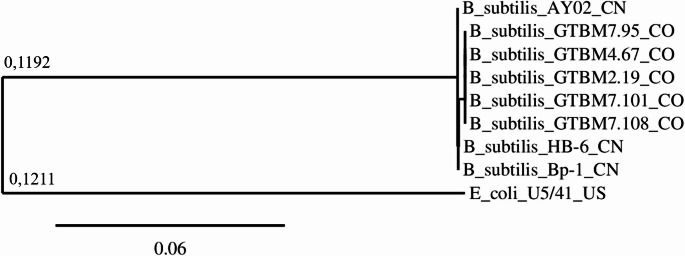
Phylogenetic tree with *Bacillus subtilis* sequences of the 16 S ribosomal gene. The evolutionary distribution was inferred with the Neighbor-joining method. The scale means a substitution of six nucleotides per 100 positions, and *Escherichia coli* US/41 was used as an outgroup in the analysis

*Bacillus subtilis* isolated from a typical cacao fermentation process from Colombia were grouped into a clade. In contrast, other *Bacillus* isolates, such as AY02, HB-6, and Bp-1 from China, were isolated from *Triticum* sp. (wheat), industrial wastewater, and swine, respectively (Fig. 5).

### Evaluation of the physical and chemical variables on micro-fermentation

**Fig. 6:**
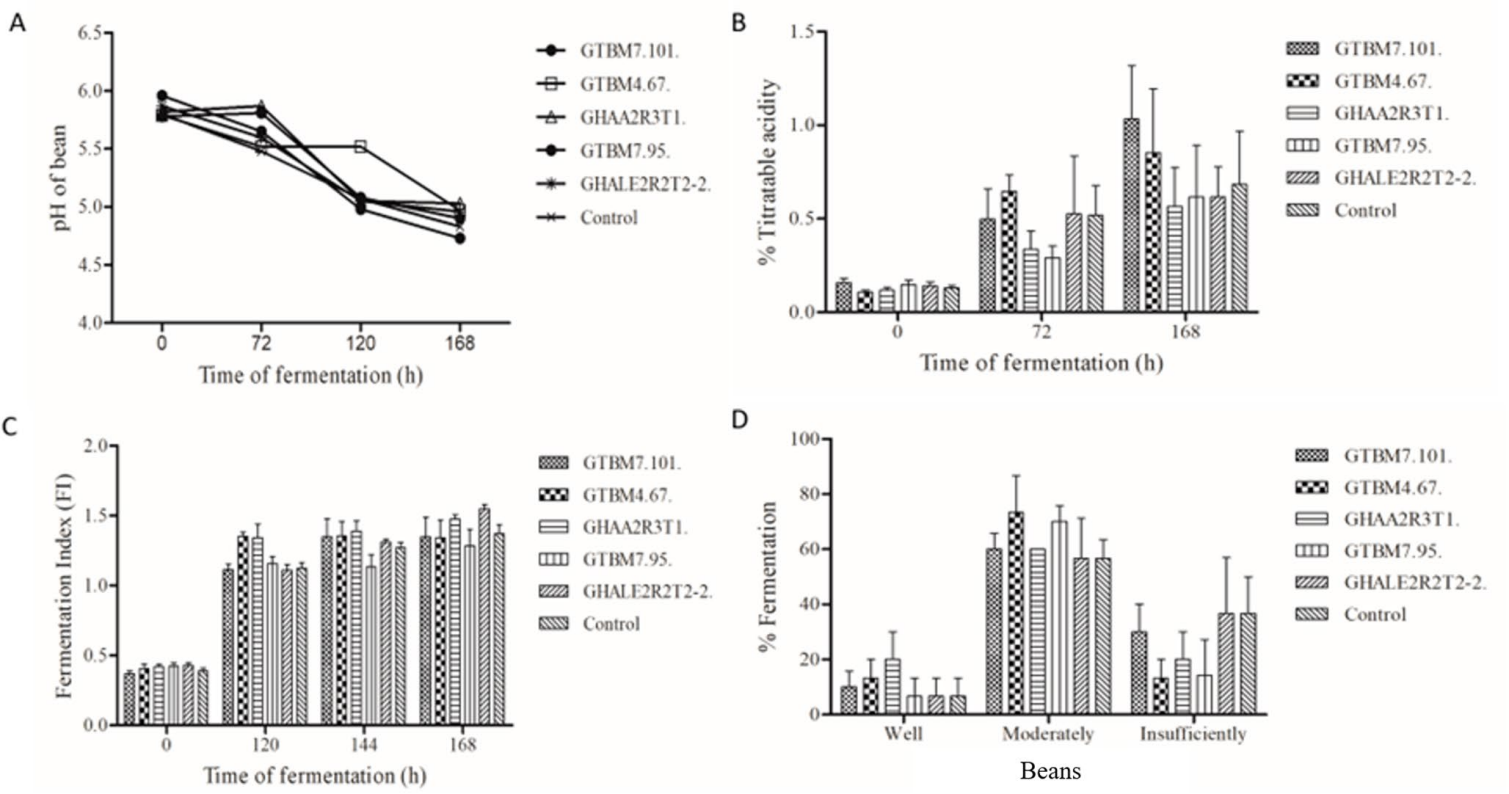
Physical and chemical variables during the fermentation process of the cacao beans. pH (**A**), titratable acidity (**B**), fermentation index (**C**), and percentage of fermentation in well-fermented, moderately-fermented, and insufficiently-fermented beans (cut test) (**D**)

The selected isolates for the starter culture were GTBM4.67, GTBM7.95, GTBM7.101, GHAA2R3T1, and GHALE2R2T2-2. The pH at the beginning of fermentation in the treatments shifted between 5.7 and 5.9, with a gradual decline as the fermentation continued, reaching values of 4.7 and 5.0. This behavior was similar in all treatments (Figs. 6.A and 6.B). However, according to the fermentation index (FI) analysis, no significant differences were found. At 168 h, GHAA2R3T1 and GHALE2R2T2-2 showed higher values, both with a FI of 1.5 (Fig. 6.C). Furthermore, the cut test on the control at 168 h showed 36% of insufficiently fermented beans subjected to micro-fermentation, while GHAA2R3T1 and GTMB4.67 showed 20% and 13% of insufficiently fermented beans, respectively (Fig. 6.D).

### Sensory profile

**Fig. 7:**
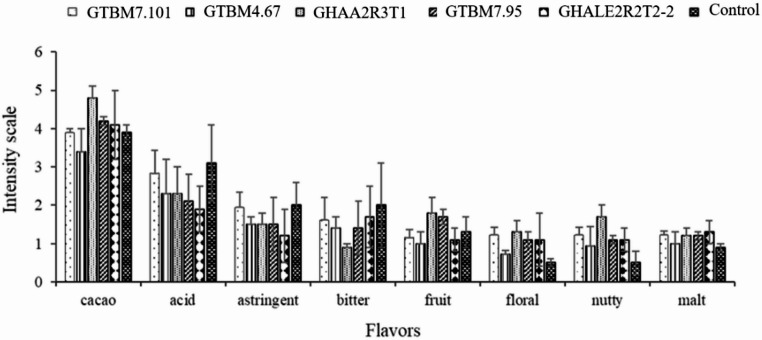
Sensory profile of the cacao beans fermented at 168 h with the addition of three *Bacillus* spp. isolates

The sensory evaluation of the micro-fermentation treatments showed that the treatment with isolate GHAA2R3T1 strongly influenced the development of the cacao flavor, with 4.8, i.e., the highest values. Bitterness and astringency were established as 0.9 and 1.5 Finally, the only isolate that developed a slightly nutty flavor was GHAA2R3T1 (Fig. 7).

### Evaluation of the physical and chemical variables on macro-fermentation

**Fig. 8:**
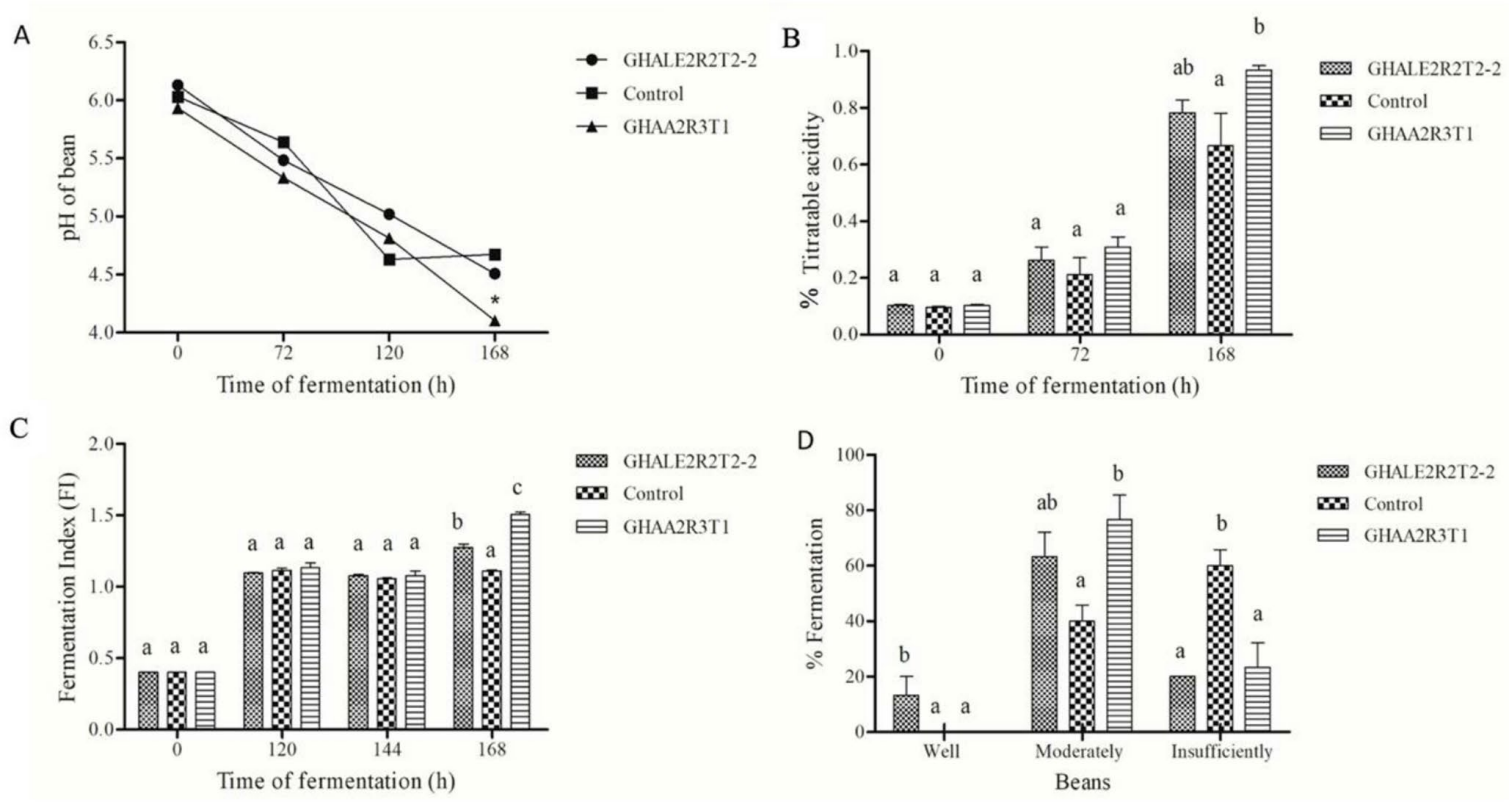
Physical and chemical variables during the fermentation process of the cacao beans. pH (**A**), titratable acidity (**B**), fermentation index (**C**), and percentage of fermentation of well-fermented, moderately-fermented, and insufficiently-fermented beans (cut test) (**D**). Bars sharing the same letter are not significantly different, whereas bars with different letters indicate significant differences according to Fisher’s LSD test (*p* < 0.05)

The macro-fermentation pH of the cacao beans showed statistical differences at 168 h, with the treatment that included GHAA2R3T1, i.e., the one with the lowest pH (4.1) (Fig. 8.A). At the same time, the treatment with GHAA2R3T1 showed the highest total acidity (0.93%) (Fig. 8.B) and the highest fermentation index (1.5) (Fig. 8.C). The cut test indicated that adding the microorganisms improved the quality of the slightly fermented beans and lowered the number of insufficiently fermented beans compared to the control (Fig. 8.D).

**Fig. 9:**
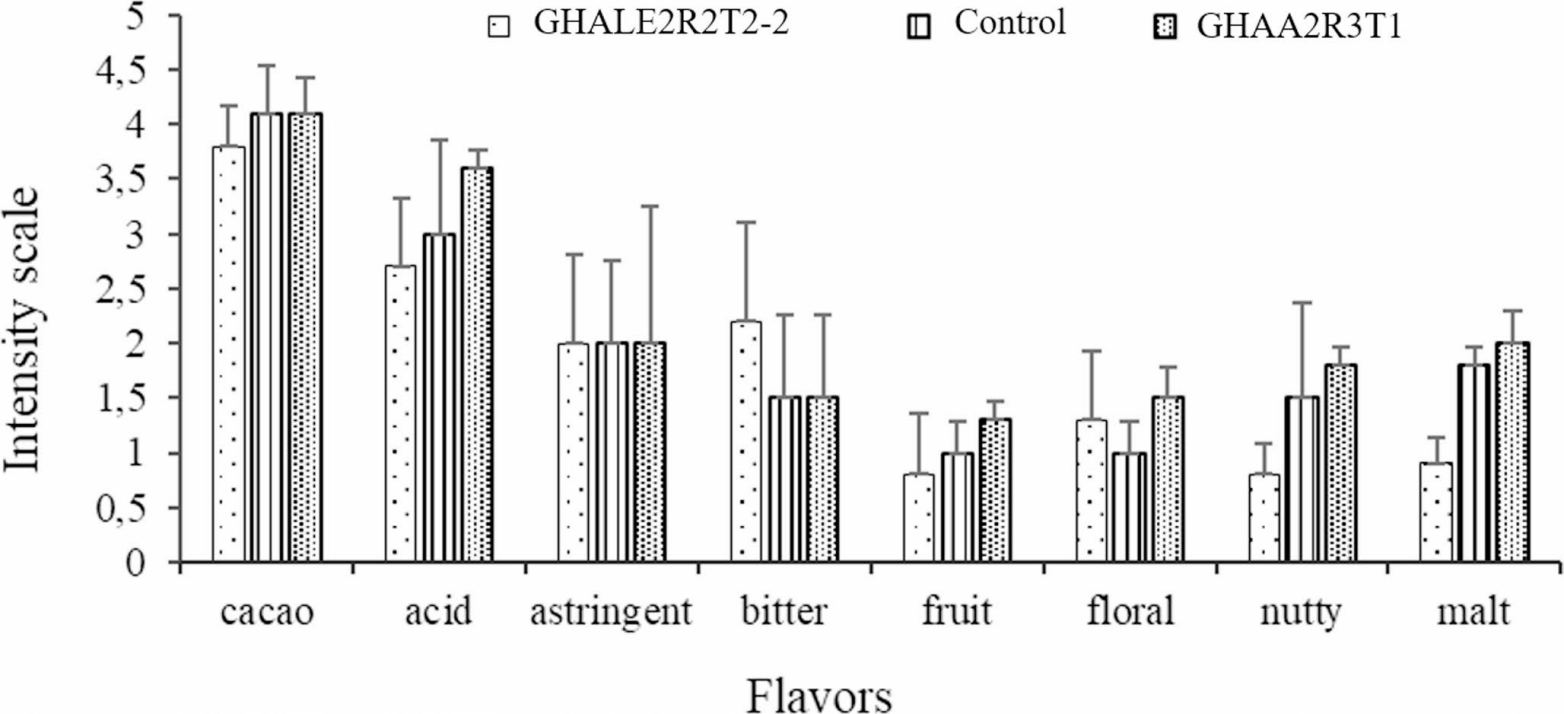
Sensory profile of the macro-fermentation with isolates GHALE2R2T2-2 and GHAA2R3T1 and the control (spontaneous fermentation)

According to the sensory analysis for the macro-fermentation samples, the acid, fruity, and nutty flavors are notable and slightly superior in the treatment with isolate GHAA2R3T1 compared to the control (Fig. 9).

## Discussion

### Selection of *Bacillus* isolate to improve the quality and safety of cacao beans

The technological properties of microorganisms must be studied prior to their use as potential starters. In our work, the GTBM4.67 isolate showed the highest growth in the medium supplemented with 10% glucose, and only four isolates (GTBM2.19, GTBM4.67, GTBM6.85, and GTBM7.95) grew in the MRS medium supplemented with 5% ethanol. The most important properties are resilience to high sugar, alcohol, and acid concentrations (Yao et al. 2017), considering that the sugar concentrations from the cacao pulp are high, ranging from 8 to 12 g/kg of saccharose, 0.097 and 0.375 g/kg of glucose and between 0.083 and 0.357 g/kg of fructose; finally, ethanol concentration can reach 0.7% (Moreira et al. 2013) and 8% (Pereira et al. 2012).

In this research, six isolates tolerated a temperature of 50 °C. The isolates were not as susceptible to high sugar concentrations, but four isolates (GTBM2.39, GTBM2.49, GTBM4.62, and GTBM7.99) did not tolerate any stress condition. For this reason, they were not used as starter cultures. In 2017, Yao et al. (2017) evaluated the growth of *Bacillus* in a culture medium enriched with 8% ethanol, obtaining similar results, as most of the *Bacillus* isolates could not survive at this concentration. These results may explain the prevalence of these microorganisms at the end of the fermentation when the ethanol concentration decreases and the temperature increases (Figueroa et al. 2019).

Additionally, the production capacity of pectinolytic and proteolytic enzymes and the citric acid decomposition were described as fundamental characteristics of a successful fermentation process (Ouattara et al. 2017). Of the 12 *Bacillus* isolates tested in the 50% Cacao Pulp Medium Agar at pH 4.8, only 60% of the microorganisms registered halos. Probably, the pulp pH inhibited the remaining 40% of the isolates. Arellano et al. (2015) evaluated the pectinase production of *Bacillus* isolates at the same pH, but no pectinolytic activity was detected. In the current study, the isolates with the biggest halos were GTBM7.101 and GHALE2R2T2-2. Several studies have indicated that *Bacillus* has high pectinolytic activity and can produce at least one type of pectinase (Ouattara et al. 2008, 2011). In assays carried out by Auta et al. (2013), the enzyme produced by *Bacillus* sp. FWS II-4 demonstrated its highest activity when incubated at 40 °C and pH 4.0. This result may confirm that pectinase can tolerate high temperatures up to 50 °C. Moreover, pectinase improves the fermentation grade in the beans (Bhumibhamun and Jinda 1997). All the *Bacillus* isolates used as starting cultures formed halos on the Milk and Casein Agar Cultures, indicating protease activity. These enzymes are essential for developing the chocolate flavor (De Souza et al. 2018).

*Bacillus* can produce from two to four proteolytic enzyme groups. The metalloproteases are endoenzymes related to phenylalanine, a molecule that can move inside the bean and be part of the internal precursors. Therefore, phenylalanine favors the development of the chocolate aroma (Castro et al. 2019). Researchers have also studied the use of proteolytic enzymes in cacao fermentations. Depending on the source of protease used to ferment the beans, there are differences in the organoleptic perception of the chocolate flavor. The flavor intensifies with an optimal pH of 4.7, a temperature of 40 °C, and adding the protease at the beginning of the third day of the process (Navia and Pazmiño 2012). Oliveira et al. (2011) used plants as sources of proteases. The author compared them to microbial proteases, and the products obtained with one of these had a favorable acceptance by the sensory evaluation panel. Finally, all the *Bacillus* isolates evaluated in Simmons Citrate Medium were positive for citrate. This result indicates that the evaluated isolates possess the metabolic capacity to utilize citrate during cacao fermentation, a characteristic considered fundamental for a successful fermentation process. Citrate is included in the metabolism as succinic acid, lactic acid, acetone, or 2,3-butanediol (Ouattara et al. 2017), precursors of malt, sweet, fruit, and chocolate aromas. Therefore, the ability of all isolates to metabolize citrate suggests their potential contribution to the formation of key aroma compounds during the fermentation process, as reported in previous studies. Ouattara et al. (2020) discovered that *Bacillus* produces high quantities of 1-butanethiol, acetone, phenethyl alcohol, and acetic acid. These metabolites are consistent with citrate consumption pathways, where 1-butanethiol and acetone generate sweet and butter-like aromas, respectively. The phenethyl alcohol, in low quantities in cacao beans, develops nutty aromas, and ethylene phenol acetate forms sweet and honey aromas (Prado and Zambrano 2015).

### Evaluation of the antifungal effect of *Bacillus* crude extracts on the mycelia growth of mycotoxin-producing fungi

The evaluation of the antifungal activity of *Bacillus* extracts registered low inhibition percentages and isolates GTBM7.101 and GHAA2R3T1 registered the highest inhibition, but only against *Aspergillus* spp 2. However, the data obtained so far are encouraging and stimulate research towards this type of interaction because of the ability of these isolates to produce a wide diversity of metabolites with antifungal activity (Rojas et al. 2017). This protective effect may be due to different mechanisms that can influence the growth of pathogens either negatively, directly, or indirectly. Einloft et al. (2017) reported the antagonistic activity of the *Bacillus* genus on different phytopathogenic fungi attributed to the production of antibiotics. Furthermore, their endospore formation feature offers them resistance to environmental changes.

### Evaluation of the effects of adding *Bacillus* isolates on the quality of cacao beans

The evaluation of isolates GTBM4.67, GTBM7.95, GTBM7.101, GHAA2R3T1, and GHALE2R2T2-2 in the microfermentation of cacao beans showed no significant differences in the physicochemical variables evaluated, including fermentation index (FI), titratable acidity, bean pH, and cut test (% fermentation). The sensory evaluation suggested that beans fermented with GHAA2R3T1 tended to exhibit a more favorable sensory profile, characterized by more pronounced aroma, nutty notes, and a more intense chocolate flavor. The CCN51 genotype was selected for this study because it is a material with organoleptic characteristics that generated rejection in specific commercial sectors (Quintana et al. 2012). For this reason, improving its sensory profile could be an advantage. Further, GHAA2R3T1 was the only isolate that obtained 20% of well-fermented beans. At the same time, the FI did not show a significant difference; however, at 168 h, GHAA2R3T1 showed higher values than the other treatments.

Similarly, in the macrofermentation (field fermentation), the treatment with the highest FI (1.5) was GHAA2R3T1, with significant differences between treatments. Bacillus megaterium GHAA2R3T1. This isolate may also have promoted the degradation of high-molecular-weight polyphenols and tannins, which is associated with the color change of beans from purple to brown and an increase in FI. Polyphenol degradation and pyrazine formation are both important processes during cacao fermentation and flavor development (Eyamo et al. 2016). Pyrazines are key aroma compounds involved in chocolate flavor, particularly contributing roasted, cocoa, and nutty notes. Their concentration can be influenced by environmental conditions, cacao genotype, harvest maturity, and processing steps (Moreira et al. 2018). Among them, tetramethylpyrazine is mainly formed during fermentation and roasting through the Maillard reaction (Hashim, 1998). Although pyrazines were not analytically quantified in this study, the intensity of cacao and nutty flavors perceived by the sensory panel in beans fermented with GHAA2R3T1 both in micro-fermentations and compared with the control suggests a potential association with pyrazine related aroma development. Furthermore, during macrofermentation, cocoa beans fermented with *B. megaterium* GHAA2R3T1 exhibited a pH of 4.1 at seven days of fermentation. This value was consistent with the sensory profile, which showed a higher intensity of acidity compared with the other two treatments. Nevertheless, higher intensities of floral, fruity, and nutty attributes were also observed, resulting in this cocoa liquor being the most preferred by the panelists.

Based on the *Bacillus* metabolism, Yao et al. (2017) suggested that these microorganisms could improve cacao quality. Ouattara et al. (2008) also recommended using *Bacillus* in the fermentation for high pectinase production and high-temperature tolerance, i.e., the normal conditions at the end of fermentation. The effect of *Bacillus subtilis* with *Pichia kudriavzevii* was tested, and a synergy relationship was obtained, showing higher ethanol production and pulp degradation when these microorganisms were added together; however, *P. kudriavzevii* inhibited the growth of *Bacillus* (Ouattara et al. 2008).

### Future perspectives and safety considerations

Despite the promising results obtained in this study, although *Bacillus megaterium* is generally regarded as a low-risk microorganism, strain-level safety evaluation was not addressed. Therefore, further studies are required to assess the safety of the GHAA2R3T1 strain, including molecular and toxicological analyses, before it can be considered for Generally Recognized as Safe (GRAS) status and industrial application. These evaluations were beyond the scope of the present study and should be addressed in future research.

## Conclusions

This study represents the first evaluation of *Bacillus* isolates as potential starter cultures for cacao fermentation in Colombia. The inclusion of *Bacillus* species during cacao bean fermentation did not result in significant differences in physicochemical parameters; however, their presence was associated with a tendency toward improved sensory attributes and a reduced incidence of fungal growth during fermentation. In particular, the participation of the *Bacillus megaterium* isolate GHAA2R3T1 was associated with more favorable flavor attributes in cacao beans of the CCN51 genotype. Overall, these findings suggest that *B. megaterium* may represent a promising biotechnological resource for further studies aimed at improving the quality and safety of cacao beans.

## Supplementary Information

Below is the link to the electronic supplementary material.

Supplementary Material 1 (DOCX 81.6 KB)

## Data Availability

Data is provided within the manuscript or supplementary information files.
